# Best single-slice measurement site for estimating visceral adipose tissue volume after weight loss in obese, Japanese men

**DOI:** 10.1186/1743-7075-9-56

**Published:** 2012-06-14

**Authors:** Rina So, Tomoaki Matsuo, Hiroyuki Sasai, Miki Eto, Takehiko Tsujimoto, Kousaku Saotome, Kiyoji Tanaka

**Affiliations:** 1 Graduate School of Comprehensive Human Sciences, University of Tsukuba, 1-1-1 Tennodai, Tsukuba, Ibaraki, 305-8577, Japan; 2 National Institutes of Health, 9000 Rockville Pike, Bethesda, MD, 20892, USA; 3 Japan Society of the Promotion of Science, Tokyo, Japan; 4 Tsukuba Medical Center Hospital, 1-3-1 Amakubo, Tsukuba, Ibaraki, 302-0127, Japan

## Abstract

**Background:**

Although the measurement site at L4–L5 for visceral adipose tissue (VAT) has been commonly accepted, some researchers suggest that additional upper sites (i.e., L1–L2 and L2–L3) are useful for estimating VAT volume. Therefore, determining the optimum measurement site remains challenging and has become important in determining VAT volume. We investigated the influence of a single-slice measurement site on the prediction of VAT volume and changes in VAT volume in obese Japanese men.

**Methods:**

Twenty-four men, aged 30–65 years with a mean BMI of 30 kg/m^2^, were included in a 12-week weight loss program. We obtained continuous T1-weighted abdominal magnetic resonance images from T9 to S1 with a 1.5-T system to measure the VAT area. These VAT areas were then summed to determine VAT volume before and after the program.

**Results:**

Single-slice images at 3–11 cm above L4–L5 had significant and high correlations with VAT volume at baseline (r = 0.94–0.97). The single-slice image with the highest correlation coefficient with respect to VAT volume was located at 5 cm above L4–L5 (r = 0.97). The highest correlation coefficient between the individual changes in VAT area and changes in VAT volume was located at 6 cm above L4–L5 (r = 0.90).

**Conclusions:**

Individual measurement sites have different abilities to estimate VAT volume and changes in VAT volume in obese Japanese men. Best zone located at 5–6 cm above L4–L5 may be a better predictor of VAT volume than the L4–L5 image in terms of both baseline and changes with weight loss.

## Introduction

Obesity is closely associated with various metabolic disorders [1]. In particular, visceral adipose tissue (VAT) accumulation, which often accompanies obesity, is associated with hypertension, dyslipidemia, type 2 diabetes, and cardiovascular disease (CVD)[2,3]. Although subcutaneous adipose tissue (SAT) also contributes to obesity-related insulin resistance and metabolic disease [4,5], most previous studies reported that VAT has a stronger association with metabolic risk factors as compared to SAT [6,7].

Recently, high-accuracy imaging techniques, such as computed tomography (CT) and magnetic resonance imaging (MRI), have attracted much attention in the estimation of abdominal adipose tissue. These techniques allow VAT and subcutaneous SAT to be easily distinguished [8]. Consecutive images, in regular succession along the spine, are required for precise measurement of abdominal adipose tissue accumulation [8,9]. Unfortunately, taking large numbers of images per patient is not practical in a clinical setting because of the increased cost and time required as the number of images and analysis required increases [10,11]. Accordingly, most researchers use a single-slice image at L4–L5 to estimate the VAT [12-16]. Recent studies [17,18] demonstrated that the most suitable site representing the total VAT volume is 5–10 cm above L4–L5. This site was better correlated with the total VAT volume than a single-slice image taken at the level of L4–L5. Additionally, several studies [17,19,20] have shown that the VAT area measured in the upper abdomen (i.e., L1–L2 or L2–L3) is a better predictor of metabolic syndrome than the most common measurement site of L4–L5.

Weight loss is associated with improvements in risk factors for chronic disease [21,22] and all-cause mortality [23]. Thus, the identification of the measurement site best representing VAT volume is particularly important for estimating the change (Δ) in VAT due to weight loss and for evaluating the effects of weight loss programs. An early study [24] with a small sample size showed different relative changes in several VAT depots (i.e., L2–L3, L3–L4, L4–L5) in response to a 4.4 kg weight reduction induced by a liquid formula diet. Another prior study [25] suggested that the single-slice VAT area with the highest correlation with Δtotal VAT volume was located at the L2–L3 level (5–6 cm above L4–L5) after two interventions (hypocaloric diet with or without resistance training).

These previous studies [24,25] were performed with Spanish and Scandinavian populations. Thus, the previous findings were unable to adequately address the possibility of racial differences. Asian populations have lower average body mass indices but higher body fat percentages than people of European ancestry [26], and Japanese have a significantly greater amount of VAT than Caucasians [27,28] even at the same levels of waist circumference. Despite emerging evidence regarding racial differences in body composition, many races and ethnic groups are still poorly represented in a clinical setting. A person’s race or ethnicity may influence the measurement site that is best for determining VAT, and to date, there are no data showing the best measurement site for predicting changes in VAT in the Japanese. Thus, the objective of this study was to investigate the relationship between single-slice MRI images and abdominal fat tissue volume. Our focus in this longitudinal study was to identify the single-slice measurement site having the strongest association with VAT volume.

## Methods

### Participants

We recruited participants through local newspaper advertisements and study flyers from nearby areas and communities surrounding our university for a 12-week weight-loss program. Initially, 78 men were recruited, and 27 were selected based on the following eligibility criteria: 1) aged 30–65 years, 2) a BMI > 25 kg/m^2^ according to the domestic obesity guideline [29] and 3) not taking any medications known to affect fat metabolism and weight regulation. In Japan, despite the fact that only 2–3% of the population has been characterized as having a BMI > 30 kg/m^2^, the prevalence of metabolic disorders is relatively high. Thus, the cutoff value of 25 kg/m^2^ for the definition of obesity for Asian populations was used [29]. Three men were unable to successfully complete the 12-week program (2, incomplete assessment, and 1, injured in daily life), and the remaining 24 men served as the study population. The characteristics of the participants are summarized in Table 1. All potential risks and procedures of the study were explained to the participants before they provided written informed consent to participate in the study. The study conformed to the principles outlined in the Declaration of Helsinki.

**Table 1:** Characteristics of study participants (n = 24)

		**Baseline**			**After**			**Δ**			**%Δ**	
Age, yr	46.8	±	4.7		−			−			−	
Height, cm	170.9	±	6.9		−			−			−	
Body weight, kg	88.8	±	14.7	80.2	±	2.9	−8.6	±	4.7*	−10.0	±	5.0*
BMI, kg/m^2^	30.4	±	4.6	27.4	±	0.9	−3.0	±	1.6*	−9.8	±	5.0*
Abdominal circumference, cm	101.7	±	10.0	93.4	±	2.3	−8.3	±	4.6*	−8.3	±	4.4*
Visceral fat												
volume, cm^3^	4859	±	1264	3676	±	331	−1182	±	705*	−26.6	±	17.6*
mass, kg	4.47	±	1.16	3.38	±	0.30	−1.09	±	0.65*	−27.4	±	14.1*
Subcutaneous fat												
volume, cm^3^	4696	±	1842	3877	±	359	−818	±	583*	−18.1	±	12.8*
mass, kg	4.32	±	1.69	3.60	±	0.33	−0.75	±	0.54*	−17.9	±	11.2*
Total energy intake, kcal/day	2303	±	648	1556	±	253	−747	±	739^*^	−32.4	±	11.4*
Total energy expenditure, kcal/day	2512	±	285	2474	±	263	−39	±	77*	−1.5	±	2.1*
Physical activity energy expenditure, kcal/day	348	±	163	349	±	132	0.7	±	65	0.2	**±**	1.8
Number of steps, steps/day	8555	±	2801	8859	±	2411	305	±	1408	3.5	**±**	5

Δ = After - Baseline.

Values are presented as mean ± standard deviation.

^*^ Significant change within group by paired *t*-test (*P* < 0.05).

### Intervention

The participants took part in a 12-week weight loss program from August 2009 to October 2009. The program was mainly comprised of weekly dietary modification sessions (90 minutes per session, 12 times in 12 weeks). Each session included lectures to help adopt a nutritionally well-balanced diet (30–90 minutes per lecture) and individual counseling by trained staff. Participant’s dietary habits were modified based on the Four-Food-Group Point Method [30], otherwise known as the Kagawa Nutrition University Diet (KNUD) (http://co-4gun.eiyo.ac.jp/English%20Version/Index1-English. html). The participants kept a daily diary in which they recorded in detail every food they ate, any transitional changes in body weight, and their health and mental conditions during the 12-week program period. At every dietary session, trained staff checked each participant’s diary as precisely as possible, adding their advice, while participants were taking part in the lectures. The staff provided face-to-face individual counseling based on the participant’s diary after the session. More detailed explanations of the program and methodology have been previously published [31].

### Anthropometric measurements

All measurements were conducted in the same order on 24 participants who completed the weight loss program at baseline and at week 12. We instructed the participants not to participate in vigorous physical activity or to consume alcohol within the 24 hour prior to the measurements. Body weight was measured to the nearest 0.1 kg using a digital scale (TBF-551; Tanita, Tokyo, Japan), and height was measured once to the nearest 0.1 cm using a wall-mounted stadiometer (YG-200; Yagami, Nagoya, Japan); for both measurement, the participants wore only underwear and were barefooted and they had been fasting for at least 8 hour. BMI was calculated as weight (in kilograms) divided by height (in meters) squared. The abdominal circumference was measured directly on the skin surface at the level of the umbilicus in the standing position. The abdominal circumference measurements were taken in duplicate to the nearest 0.1 cm, and the averaged value was used for analysis.

### Magnetic resonance imaging

MRI measurement was performed one week before the program and after the program. We instructed the participants not to wear any metal objects during the MRI measurements and to refrain from eating or drinking for 2 hour prior to the measurement. Abdominal multiple-slice MRI scans were performed by using a 1.5-T system (Siemens Magnetom Avanto syngo MR B15, Siemens, Erlangen, Germany). All participants were T1-weighted image scanned with a body coil. Imaging parameters included a spin-echo sequence with a 379-ms repetition time, an 11-ms echo time, a 48-cm field of view and a 256 × 256 matrix. The participants were instructed to lie in the magnet in a supine position with the arms extended above the head. A breath-hold sequence was used to minimize the effects of respiratory motion on the images. While scanning, participants had 4 breath-hold phases (1 phase took 25 seconds). The slice thickness was 10 mm, and images were obtained from the bottom of T10 to the top of S1. Image locations were defined relative to the common anatomical landmark of the fourth lumbar (L4) and fifth lumbar (L5) intervertebral space (L4–L5). We chose our range for evaluation according to a standard anatomy textbook.

Depending on the height of the person, this resulted in a total of 27–37 axial image per person and average 35 axial images. To precisely compare individual image data, we determined our analyses to the set of images with no missing values for all participants. This protocol is generally consistent with those used in other studies [25,32]. The average total scanning time was 15 minutes for each participant, and all scanning was performed by the same radiological instrument technician. The images were retrieved from the scanner according to a DICOM (Digital Imaging and Communications in Medicine) protocol. After image acquisition, the segmentation and quantification of SAT and VAT were performed by an experienced technician. The VAT was defined as the adipose tissue in the region enclosed by the inner aspect of the abdominal wall and the anterior margin of the vertebral body including the intra-abdominal cavity. The SAT was defined the adipose tissue in the anterior margin of the vertebral body and in the area enclosed by the innermost aspect of the abdominal muscle wall and the skin surface. Each image was segmented and quantified using image analysis software (Slice-O-Matic, Tomovision Inc, Montreal, Canada). The model and method employed to segment the various tissues is fully described and illustrated elsewhere [33]. The areas (cm^2^) of SAT and VAT in each image were computed automatically by summing the adipose tissue pixels and multiplying the number of pixels by the individual pixel size. The technical errors for 2 readings of the same scan by the same observer for SAT and VAT volumes in our laboratory were 1.23% and 2.27%, respectively (n = 82).

### Energy intake and physical activity

All participants also kept daily food diaries during the 12-week intervention period and learned about daily nutrition through weekly lectures and counseling by the dietitian. To determine dietary intake prior to starting the intervention, to determine dietary intake, 3-day weighed dietary records were conducted on randomly selected days at baseline and at week 10. Dietary intake was analyzed using commercially available software (Excel Eiyoukun version 4.0, Kenpakusya, Tokyo, Japan) [34]. The total energy expenditure (TEE), physical activity energy expenditure and number of steps were assessed by a validated uniaxial accelerometer (Lifecorder, Suzuken Co. Ltd., Nagoya, Japan). The accelerometer was firmly attached to the participant’s clothing (belt or waistband) during all waking hours beginning 2 weeks before starting the 12-week program and continuing though until its completion. Detailed descriptions of the accelerometer have been published elsewhere [35].

### Statistical analysis

The necessary sample size based on a previous study (r = 0.99) [36], and the needed sample size is 42 when the power is 0.95. Values are expressed as the mean ± SD. Paired Student’s *t-*tests were used to compare values before and after the program. Images ranged from 20 cm above L4–L5 (+20 cm) to 3 cm below L4–L5 (−3 cm), which yielded 24 images per participant. Volumetric units (cm^3^) were used throughout, unless mass (kg) was calculated (using the conversion equation of 1 liter adipose tissue = 0.923 kg). Pearson’s correlation analyses were then applied to ascertain the association of the single-slice image area with respect to the VAT or SAT volumes. We compared each correlation coefficient with the coefficient of L4–L5 using Fisher’s Z transformation method [37]. The data were analyzed with the Statistical Analysis System (SAS), version 9.2 (SAS Institute Inc, Cary, NC, USA). We considered a *p* value less than 0.05 to be statistically significant.

## Results

### Changes in body composition and energy balance

The average attendance at the dietary sessions (in total 12 times) was 71.7%, ranging from 60 to 83%. Baseline characteristics and changes in these characteristics with weight loss are given in Table 1. The values of anthropometrics characteristics were significantly lower after the program. Significant reductions were observed in total energy intake and TEE during the program (Table 1). We observed no significant changes in PAEE and number of steps over the 12-week period. Table 2 describes individual single-slice areas at baseline and after the program.

**Table 2:** comparison of single-slice image areas at baseline, after and change

**Level (cm)**	**VAT**			**SAT**		
	**Baseline** (**cm**^**2**^)	**After** (**cm**^**2**^)	Δ (**cm**^**2**^)	**Baseline** (**cm**^2^)	**After** (**cm**^**2**^)	Δ (**cm**^**2**^)
20	95 ± 47	65 ± 40	−30 ± 37^*^	138 ± 63	126 ± 65	−12 ± 19^*^
19	123 ± 61	82 ± 54	−41 ± 47^*^	136 ± 62	123 ± 62	−14 ± 20^*^
18	144 ± 65	101 ± 63	−43 ± 52^*^	136 ± 62	121 ± 62	−16 ± 20^*^
17	160 ± 59	116 ± 71	−44 ± 57^*^	136 ± 62	118 ± 60	−18 ± 20^*^
16	175 ± 56	132 ± 75	−43 ± 52^*^	137 ± 64	117 ± 59	−20 ± 21^*^
15	197 ± 58	150 ± 77	−48 ± 48^*^	140 ± 66	118 ± 59	−22 ± 22^*^
14	222 ± 58	168 ± 77	−54 ± 51^*^	144 ± 67	119 ± 59	−24 ± 22^*^
13	233 ± 53	181 ± 79	−52 ± 48^*^	148 ± 69	122 ± 60	−27 ± 23^*^
12	241 ± 55	189 ± 82	−52 ± 50^*^	156 ± 71	127 ± 62	−29 ± 24^*^
11	243 ± 58	194 ± 82	−49 ± 47^*^	163 ± 73	133 ± 65	−29 ± 25^*^
10	255 ± 71	201 ± 88	−54 ± 43^*^	176 ± 75	142 ± 68	−34 ± 26^*^
9	258 ± 69	197 ± 86	−61 ± 43^*^	187 ± 76	151 ± 70	−36 ± 26^*^
8	250 ± 68	185 ± 82	−64 ± 36^*^	194 ± 75	159 ± 74	−35 ± 26^*^
7	241 ± 74	186 ± 85	−55 ± 38^*^	202 ± 75	168 ± 78	−33 ± 26^*^
6	241 ± 74	184 ± 91	−57 ± 37^*^	210 ± 77	176 ± 82	−34 ± 28^*^
5	240 ± 73	182 ± 89	−58 ± 39^*^	220 ± 78	183 ± 84	−37 ± 30^*^
4	234 ± 69	179 ± 86	−55 ± 43^*^	229 ± 79	190 ± 87	−40 ± 31^*^
3	227 ± 67	172 ± 81	−54 ± 43^*^	236 ± 81	195 ± 89	−41 ± 31^*^
2	215 ± 63	165 ± 70	−50 ± 37^*^	243 ± 86	202 ± 90	−41 ± 33^*^
1	205 ± 63	156 ± 67	−48 ± 36^*^	254 ± 93	209 ± 91	−45 ± 30^*^
0	193 ± 56	148 ± 63	−44 ± 38^*^	266 ± 98	217 ± 91	−50 ± 34^*^
−1	174 ± 55	129 ± 51	−45 ± 39^*^	277 ± 106	220 ± 91	−57 ± 43^*^
−2	154 ± 52	111 ± 47	−42 ± 43^*^	286 ± 114	221 ± 91	−65 ± 57^*^
−3	141 ± 45	106 ± 41	−35 ± 37^*^	283 ± 115	221 ± 89	−62 ± 55^*^

∆ = After - Baseline.

Values are presented as mean ± standard deviation.

^*^ Significant change within group by paired *t*-test (*P* < 0.05).

### Relationships between single-slice image areas and VAT and SAT volumes

**Figure 1:**
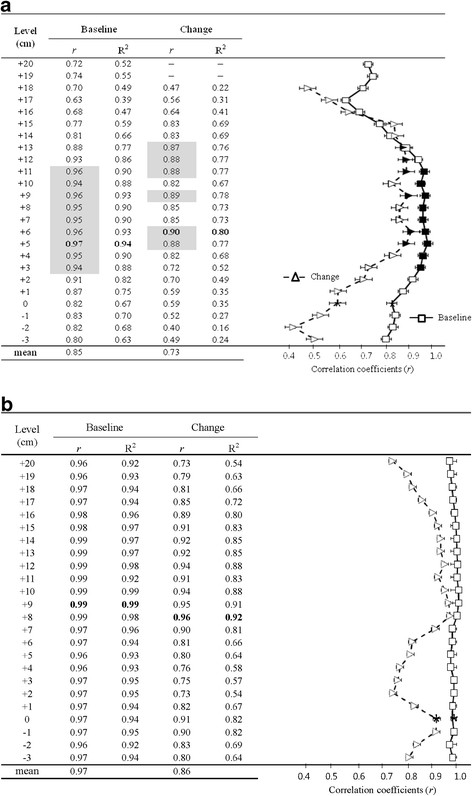
**The variation in the correlations between the VAT (a) or SAT (b) volumes and the single-slice image areas (right) (Baseline: (□), change: (Δ)); shaded symbols (■ and ▲) indicate significantly greater correlations with volume than that for L4–L5 (Data not shown for 19–20 cm above L4–L5 in VAT, for which there were no significant correlations).** (*) indicates the level of L4–L5. Measurement locations were from 3 cm below L4–L5 to 20 cm above L4–L5. n = 24. Bold indicates the highest correlation coefficient. MRI, magnetic resonance imaging. Δ, changes.

Figure 1 shows the relationships between the areas obtained using the single-slice images and the VAT and SAT volumes. For reference, the site marked “0” is the L4–L5 image, and images collected at 1-cm intervals above that point (toward the head) are labeled +1 to +20. Images collected at 1 cm intervals below that point (toward the feet) are labeled −1 to −3. Before the program, the correlations between the individual single-slice image areas and the VAT and SAT volumes were generally high (VAT: mean *r* = 0.85, SAT: mean *r* = 0.97). The single-slice image with the highest correlation coefficient with respect to VAT volume was located at 5 cm above L4–L5 (*r* = 0.97). The correlation coefficients for images 3–11 cm above L4–L5 were significantly higher than that for the L4–L5 image by Z transformation. For SAT, there were no single-slice images that exhibited significantly higher correlation coefficients than the L4–L5 image. In addition, the variation in the correlations at baseline was greater for VAT than SAT (VAT: 0.68–0.97, SAT: 0.96–0.99).

### Relationships between δsingle-slice image areas and ΔVAT and ΔSAT volumes

The correlation coefficients between the corresponding individual Δsingle-slice image areas and the ΔVAT and ΔSAT volumes after the program were generally lower (VAT: mean *r* = 0.68, SAT: mean *r* = 0.86) than those at baseline (Figure 1). Significant relationships between individual Δsingle-slice image areas and the ΔVAT volume were observed for all images except for those 19–20 cm above L4–L5. The highest correlation coefficient between the individual ΔVAT area and the ΔVAT volume was located at 6 cm above L4–L5 (*r* = 0.90). In addition, there were significantly greater correlation coefficients for the images at 5 cm, 9 cm and 11–13 cm above L4–L5 than that at L4–L5.

On the other hand, the correlation coefficients between the individual ΔSAT area and the ΔSAT volume were generally higher than those for the ΔVAT. However, no significant differences in the correlation coefficients were observed between the L4–L5 area and each individual area. Meanwhile, the variation in the correlation coefficients was greater for the ΔVAT in response to weight loss (VAT: *r* = 0.47–0.90, SAT: 0.73–0.96). In comparison with the ΔVAT, the ΔSAT area at L4–L5 correlated significantly better with the ΔSAT volume (*r* = 0.91), and the correlation was significantly stronger than the mean (*r* = 0.86).

## Discussion

The primary findings of this study were that single-slice image areas taken at different measurement sites have different associations with VAT volume and changes in VAT volume. Furthermore, images taken at 5–6 cm above L4–L5 had a better association with VAT volume and its changes in obese, Japanese men than images taken at L4–L5. The disc level that corresponds to 5–6 cm above L4–L5 was close to L2–L3 in 10.8% of participants and precisely at L2–L3 in 89.2% of participants in our study. Furthermore, we found that the relations between single-slice image changes at various measurement sites and the volume change were much more variable for VAT than for SAT.

Before the program, the image located at 5 cm above the L4–L5 image explained 94% of the variance in VAT volume (Figure 2), a correlation that was significantly greater than that for L4–L5 (67%). Our results are in agreement with those of recent studies [25,36] suggesting that positions higher on the abdomen (L2–L3 site and/or images located ~5–10 cm above the L4–L5) are better choices for assessing the VAT (L2–L3: 0.95–0.96, L4–L5: 0.81–0.89).

Dermerath et al.[38] reported that, in 820 subjects, single-slice images taken 6 cm above L4–L5 (near the L3 vertebra) accurately predicted the total VAT volume in both black and white adults (*r* = 0.98). Similarly, Kuk et al. [19] indicated that the VAT areas at L1–L2 and L2–L3 (e.g., 8–9 cm and 5–6 cm above L4–L5) were more strongly correlated with VAT volume than the VAT areas obtained at all other anatomical landmarks (*r* = 0.98 for L1–L2 and L2–L3). In the present study, we demonstrated for the first time the relationships between single-slice image areas relative to L4–L5 and VAT volume in obese Japanese men. These findings suggest that, although Asians have different body build and frame size than Caucasians [39] and Europeans [40], there may not be remarkable differences among races with respect to abdominal adipose tissue distribution patterns.

As for the ΔVAT volume in response to weight loss, the influence of the measurement site on ΔVAT estimation became greater after weight loss (Figure 2(a)). The image located at 6 cm above L4–L5 explained 80% (r = 0.90) of the variance in the ΔVAT volume, but the image located at L4–L5 explained only 35% (r = 0.59) of the variance. This result suggests that estimation of the ΔVAT volume using a single-slice images required the use if an image taken at 5–6 cm above the L4–L5 (the highest correlation coefficients at baseline and changes). In agreement with our findings, Kamel et al. [41] reported that, in 44 subjects (23 women and 21 men), a measurement site 5–10 cm above L4–L5 showed the best correlation (women; *r* = 0.92, men; *r* = 0.94) with the changes in VAT volume after a program period (weight loss treatment resulted in a comparable mean weight loss of 9.5 kg in men). On the other hand, in a longitudinal study on obese, postmenopausal women (6-month exercise intervention) Kuk et al. [42] examined the influence that measurement site may have on the association between VAT and SAT volumes and the metabolic syndrome. They reported that VAT at L1–L2 and L2–L3 were significantly stronger correlates of VAT volume as compared to L4–L5 in both baseline levels and changes that occurred with the intervention. As for correlation between VAT areas and VAT volume in the present study, we found a similar tendency. These findings support the statement that a single-slice image at L4–L5 can lead to a misestimation of the VAT accumulation and changes in VAT accumulation that occur with an intervention, which would likely lead to a misunderstanding of the clinical effects of the intervention. Kuk et al. also concluded that, although measurement sites have an impact on the prediction of VAT volume, this does not translate into an improved prediction for the metabolic syndrome. However, the Kuk study’s conclusions on this matter need careful interpretation because there was little weight loss (−0.44 kg over 6-months) during the intervention and no data on the energy balance (intake and expenditure).

In our study, single-slice images taken 5–6 cm above L4–L5 had the highest correlation with VAT volume and changes in VAT volume. The reasons why such images have a strong relationship with VAT volume remains unclear, and this should be investigated in the future. However, the single-slice image having the highest association with VAT volume was not the slice having the largest absolute VAT area and largest change in VAT area secondary to the weight loss intervention. This observation suggests the need to think about a metabolic difference not a quantity difference. Shen et al. [36] reported a possible explanation pertaining to VAT’s 2 compartments: intraperitoneal and extraperitoneal adipose tissue (IPAT and EPAT, respectively [33]). Whereas EPAT components serve primarily as mechanical cushions for organs, IPAT depots have high metabolic activity, and thus, they may account for a large proportion of the intersubject variation in observed VAT volume [36]. Consequently, a slice that contains mostly IPAT may show the highest correlation with VAT volume even though the slice does not have the largest VAT area. Our study found that the single-slice areas 5–6 cm above L4–L5 had larger decreases in VAT volume after an intervention than areas at L4–L5, and these results support previous studies [43,44]. This observation supports the hypothesis that slices above L4–L5 contain more metabolically active VAT and it is also consistent with the observation that IPAT is located primarily in the upper abdomen [44]. Ross et al. [45] reported that VAT reduction in the upper abdomen (+5 cm, +10 cm, +15 cm above L4–L5) was greater than that observed in the lower abdomen (−5 cm below L4–L5). This study examined VAT reduction by the different weight loss method (diet and aerobic exercise vs. diet and resistance exercise). This suggests possibility of higher metabolic activity of IPAT in upper abdomen in both methods.

According to these findings, the use of conventional L4–L5 single-slice images can lead to misestimating the distribution of abdominal adipose tissue and the changes in abdominal adipose tissue with weight loss, which may lead to an over- or underestimation of weight-loss effects. Therefore, it is important to determine the best measurement site to evaluate the effects of weight loss. The advantage of the present study was that this was the first study to show the distribution patterns of abdominal adipose tissue changes and their relations to VAT or SAT volumes exclusively in obese Japanese men. Racial differences in VAT have been reported between Asian and European populations, indicating that the former have relatively greater amounts of VAT than the latter even at the same level of waist circumference [28]. Moreover, a recent study suggests that, in men, racial differences in VAT varied significantly depending on measurement location [46]. However, until now, possible racial differences in the pattern of VAT deposition across different anatomical locations have not been clearly understood [47]. Although we did not make direct racial comparisons in our study, by using the same analytical methods as previous studies [32,38] and having similar results, we found there may not be many substantial differences between races on these participants.

There are several noteworthy limitations of this study. First, the sample size was relatively small. An insufficient sample size may be concomitant with type II error. Second, the present study included only obese Japanese men; thus, our results should be applied cautiously to women and non-obese individuals. Future studies are needed to determine whether changes in adipose tissue distribution can be modified by demographic variables such as sex and age. Moreover, the variability of the association between the measurement site and metabolic risk factors (i.e., glucose, triglyceride, and HDL and LDL cholesterol levels and blood pressure) should be investigated in Japanese. Finally, although we used that VAT volume in intra-abdominal, we did not include pelvic bottom. So, our result should be interpreted with caution in terms of VAT volume definition.

In conclusion, our hypothesis was supported by demonstrating that the individual measurement sites have different abilities to estimate VAT volume and changes in VAT volume in obese Japanese men. The findings of the present study showed that located at 5–6 cm above L4–L5 were optimal zone have highest association with VAT volume both at baseline and for the changes occurring as the result of the program. As for SAT, more caudal slices provided better association with subcutaneous fat, not only at baseline but for the changes in the SAT. Taking our data as a whole, our findings showed that the measurement site should be taken into consideration to accurately estimate VAT volume before and changes resulting from weight loss. Optimal measurement site of abdominal adipose tissue will serve as an important tool in the analysis of effects resulting from lifestyle interventions such as diet or exercise. Estimating not only the reduction but also the redistribution of VAT by MRI might be helpful for the search for individual predictive factors for potential metabolic diseases in clinical study and field. However, for accurate determination of abdominal adipose tissue and intervention effect comparison, we need more study for multiple-slices in abdominal region.

## Competing interests

The authors declare no conflict of interest.

## Authors’ contributions

RS was designed study and interpretation of data in addition to drafting the manuscript; TM and HS was instrumental in the study`s inception, design and approval while providing critical analysis of data interpretation and manuscript review. ME and TT made significant contributions to the acquisition of the data. The final manuscript have been read and approved by all authors.
